# Wood Recognition Using Image Texture Features

**DOI:** 10.1371/journal.pone.0076101

**Published:** 2013-10-11

**Authors:** Hang-jun Wang, Guang-qun Zhang, Heng-nian Qi

**Affiliations:** 1 College of Tianmu, Zhejiang A&F University, Lin'an, China; 2 Hefei Institute of Intelligent Machines, Chinese Academy of Science, Hefei, China; 3 Department of Automation, University of Science and Technology of China, Hefei, China; 4 School of Information and Technology, Zhejiang A&F University, Lin'an, China; Pennsylvania State University, United States of America

## Abstract

Inspired by theories of higher local order autocorrelation (HLAC), this paper presents a simple, novel, yet very powerful approach for wood recognition. The method is suitable for wood database applications, which are of great importance in wood related industries and administrations. At the feature extraction stage, a set of features is extracted from *Mask Matching Image* (MMI). The MMI features preserve the mask matching information gathered from the HLAC methods. The texture information in the image can then be accurately extracted from the statistical and geometrical features. In particular, richer information and enhanced discriminative power is achieved through the *length histogram*, a new histogram that embodies the width and height histograms. The performance of the proposed approach is compared to the state-of-the-art HLAC approaches using the wood stereogram dataset *ZAFU WS 24*. By conducting extensive experiments on *ZAFU WS 24*, we show that our approach significantly improves the classification accuracy.

## Introduction

As is well known, wood is a hard, fibrous structural tissue that constitutes the stems and roots of forest trees. Forests contain roughly 90 percent of the world's terrestrial biodiversity. They preserve the integrity of this biodiversity by storing carbon, regulating the planetary climate, purifying water and mitigating natural hazards such as floods [1]. In addition, wood renews itself by extracting energy from the sun, in a continuous sustainable cycle [2]. Humans have used wood for many purposes over the millennia, primarily as a fuel or to construct items of civilization such as houses, tools, weapons, furniture, packaging, artworks, and paper. Studies have shown that manufacturing from wood uses less energy and results in less air and water pollution than manufacturing from steel and concrete. Because the features and characteristics of timbers (including appearance, price, physical and chemical properties) vary widely, classifying wood types is an important practical problem with direct industrial applications. Wood analyses assist the furniture industry, wooden panel production, and even archeology, where they are crucial in identifying fraud [3]. However, wood species are difficult to classify correctly because wood compositions are complex, and existing species are highly diverse [4].

Currently, wood recognition relies excessively on experts, who base their judgment on readily visible characteristics such as color, odor, density, presence of pitch, or grain pattern. However, wood experts are not always available, and the accuracy of classification largely depends on the operator's experience and attention. Thus, an automated wood recognition method that accurately classify wood types from images is urgently required.

Wood is a heterogeneous, hygroscopic, cellular and anisotropic material. The cell walls of wood tissues are composed of micro-fibrils of cellulose and hemicellulose impregnated with lignin [5]. While most softwood species comprise tracheids cells, the structure of hardwoods is more complex, with features such as vessels (pores), wood rays, fiber, parenchyma and growth ring [6]. The characteristics and arrangement of these fibrous cells affects the strength, appearance, permeability, resistance to decay, and many other properties of the wood. Several researchers have used these wood characteristics to distinguish among wood species. For example, Piuri, *et al*. (2010) [7] classified wood types from features revealed in fluorescence spectra. Javier, *et al*. (2011) [8] used the thermograms curves obtained by thermogravimetric analysis (TG) and differential scanning calorimetry (DSC) to classify different wood species. Rojas, *et al*. (2011) [9] proposed a technique called stress-wave sounds to obtain information suitable for identification and classification of wood samples.

**Figure 1:**
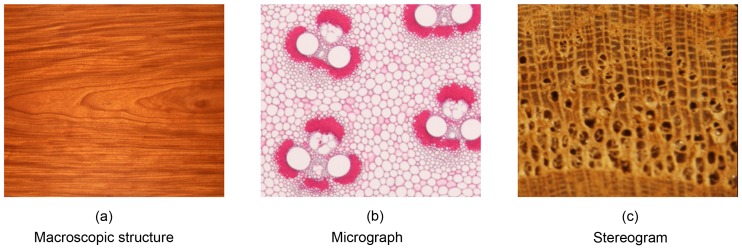
The three types of images used in computer-aided wood recognition.

However, all of these methods have given up the traditional way of recognizing wood species, which are based on wood anatomical features recognized by experts. Currently, image based methods have retained their popularity in computer-aided wood recognition research because they can integrate all known aspects of wood anatomy. Generally, there are three kinds of images can be used in wood recognition, which are macroscopic structure, micrograph and stereogram, as shown in Fig. 1. Since the appearance of each tree species is unique, the computer may be able to identify tree species directly from wood images. The International Association of Wood Anatomists (IAWA) has published a list of microscopic features for softwood and hardwood identification [10], [11]. Some of the listed features can be unambiguously assigned to a species by a computer [5], [6].

This idea has inspired researchers to develop computational techniques for classifying and recognizing wood species. These methods firstly extract semantic features, such as pores, wood rays, fiber, parenchyma, and growth rings. For example, Pan (2012) and Abhirup (2011) proposed a wood classification system based on the pores in microscopic images [12], [13]. Wang (2010) proposed a method to extract tree-rings for obtaining anatomical features such as spring or summer growth [14]. However, the success of these methods relies on correct image segmentation, which remains an elusive goal in computer vision field so far [15]. Until this problem is solved, the applicability of these methods remains limited.

To avoid the imperfection caused by image segmentation, recent studies [16]–[19] have focused on texture features. Texture analysis can extract the attributes or features from an image that differentiate one species from another. So, various feature extraction and classification methods, such as local binary pattern (LBP) [20], scale-invariant feature transform (SIFT) [21], have been proposed in the past several years for the purpose of texture analysis. More recently, methods based on higher order local autocorrelation (HLAC) have gained popularity in texture analysis. HLAC features, first proposed by Otsu [22], are derived from autocorrelation features identified from higher-order statistics (HOS) [23]. For practical computation, the original HLAC features were restricted to the second order case, represented by 25 mask patterns within a 3×3 displacement region. Many extensions to the original HLAC method have since been proposed.

Firstly, HLAC based methods with scale and rotation invariant are obtained by modifying the autocorrelation function [24], introducing multi-scale space theory [25]–[27], and Log-polar image [28]. Then, to extract more detailed information from image, later researchers altered the image functions that participate in the autocorrelation operation. They differ considerably from the early HLAC methods, in which features are extracted only from binary images. For example, Matsukawa and Kurita (2009) introduced Probability Higher-order Local Auto-Correlations (PHLAC) based on probability images [29], which extends bag-of-features technique in scene classification [30], [31]. Kobayashi and Otsu (2008) proposed Gradient Local Auto-Correlations (GLAC) and Normal Local Auto-Correlation (NLAC) [32], gradient-based techniques that utilizes the second statistics of spatial and orientation autocorrelations to discriminate specimens more powerfully than standard histogram based methods.

Generally, the local information in HLAC features is collected by counting the matching number while the mask pattern scans the image. Increasing the size and number of mask patterns enables more detailed local information. For example, applying 8-order HLAC, Toyoda and Hasegawa achieved 223 dimensions within a 3×3 displacement region [33], [34]. However, although larger displacement regions gain more useful features, the benefits of this approach are offset by high computational cost. Matsukawa and Kurita (2010) obtained larger mask patterns by varying the spatial interval among the reference points, which make the features become robust against smaller spatial difference and noise [35].

Since the above-mentioned HLAC methods count the number of matches between image and mask, they cannot avert the increasing complexity caused by larger displacement and higher order. In this paper, we propose a novel HLAC-based method called *Mask Matching Image* (MMI) method, that retains the status of template matching while acquiring more local autocorrelation information than existing methods. In the MMI, various statistical and geometric features can be defined. Unlike previously proposed HLAC methods, which use a single feature for classification purposes (i.e., the number of image/mask matches), our proposed MMI method permits higher-order data over a larger displacement, thereby enabling a stronger classification capability. Moreover, since the MMI features can be obtained at low computational cost, the method can be implemented in hardware and deployed in practical application systems.

Unlike the majority of wood recognition methods, our method relies on wood stereogram images. Although the extraction of color and texture features from macroscopic images demands few hardware requirement [3], macroscopic information may be insufficient for identifying a wide range of wood speices. For instance, pores of diameter less than 100 µm can be observed only under a microscope [36]. However, the steps of wood slicing involved in microscopic image prepration (preliminary preparation, softening and embedding, sectioning, and staining) are quite complex [37]. Since stereogram images are obtained directly from the stereoscope without requiring wood slicing, stereogrammatry can avoid these problems.

## Materials and Methods

### 2.1 Wood Stereogram Dataset

**Figure 2:**
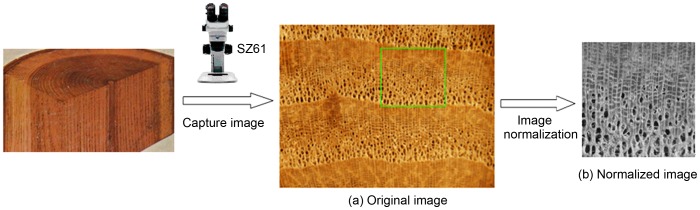
Process of acquiring, selecting and normalizing a single wood sample in the *ZAFU-WS 24* wood dataset.

To assist wood recognition research, we have compiled a wood stereogram dataset, *ZAFU WS 24* (freely downloaded from http://home.ustc.edu.cn/~hangjunw for scientific research purpose). This dataset contains stereograms of twenty four wood species located in the Zhejiang A&F University, and wildly distributed throughout Zhejiang Province, China. Stereogram images of cross section surfaces of wood samples were captured with an OLYMPUS SZ61TRC stereo-microscope and a MD50 digital imaging system. 20 images were collected for each wood species; thus, the *ZAFU WS 24* dataset contains 480 separate stereograms. Furthermore, assuming that a rectangle region between the tree growth rings was imaged for each sample, these regions can be scaled to 100×100 pixels and saved as 256 gray-level images. The requirement for selecting certain region is that the corresponding height should be exactly one growth ring, and its picture quality is high (excluding fractures, scratches and other flaws of a non-timber nature). The process of acquiring, selecting and normalizing a single image from our dataset is illustrated in Fig. 2.

**Figure 3:**
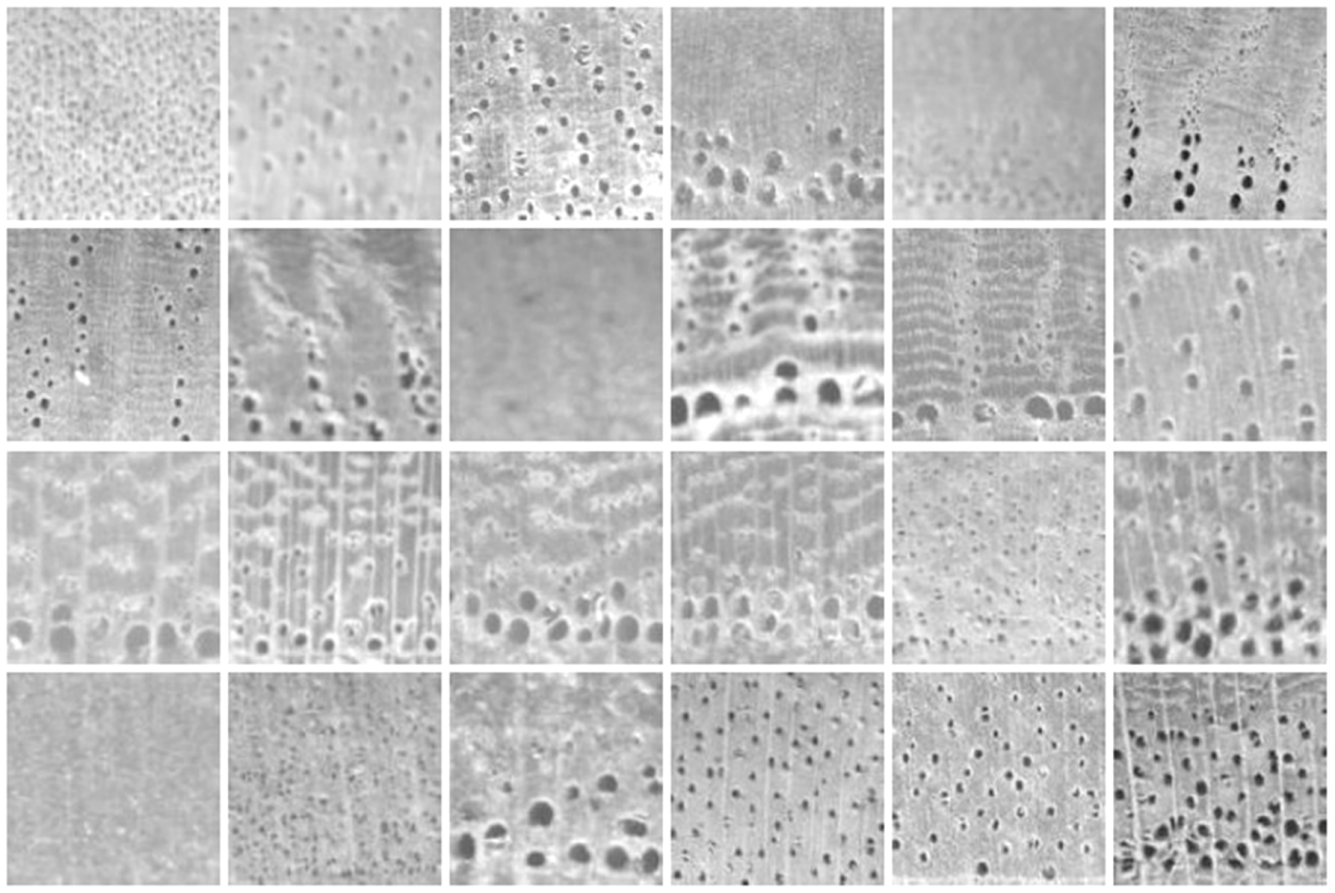
Texture images from *ZAFU WS 24* dataset used in the experiments. From left to right and top to bottom: Salix wilsonii Seem, Juglans cathayensis ver. Formasana, Juglans regia Linn, Castanea henryi (Skam) Rehd. et Wils, Castanea seguinii Dode, Castanopsis fordii Hance, Castanopsis tibetana Hance, Castanopsis sclerophylla (Lindl.)Schott, Fagus lucida Rehd. et Wils, Quercus acutissima Carruth, Quercus variabilis Blume, Aphananthe aspera Planch, Celtis biondii Pamp, Celtis bungeana Bl., Ulmus changii Cheng, Ulmus parvifolia Jacq, Litsea cubeba (Lour.) Pers, Sassafras tsumu, Photinia prunifolia (Hook. et Arn.) Lindl, Padus racemosa, Evodia fargesii Dode, z.ailanthoides Sieb. et Zucc, Toxicodendron succedaneum (Linn.) O.Kuntze, Meliosma flexuosa Pamp.

**Figure 4:**
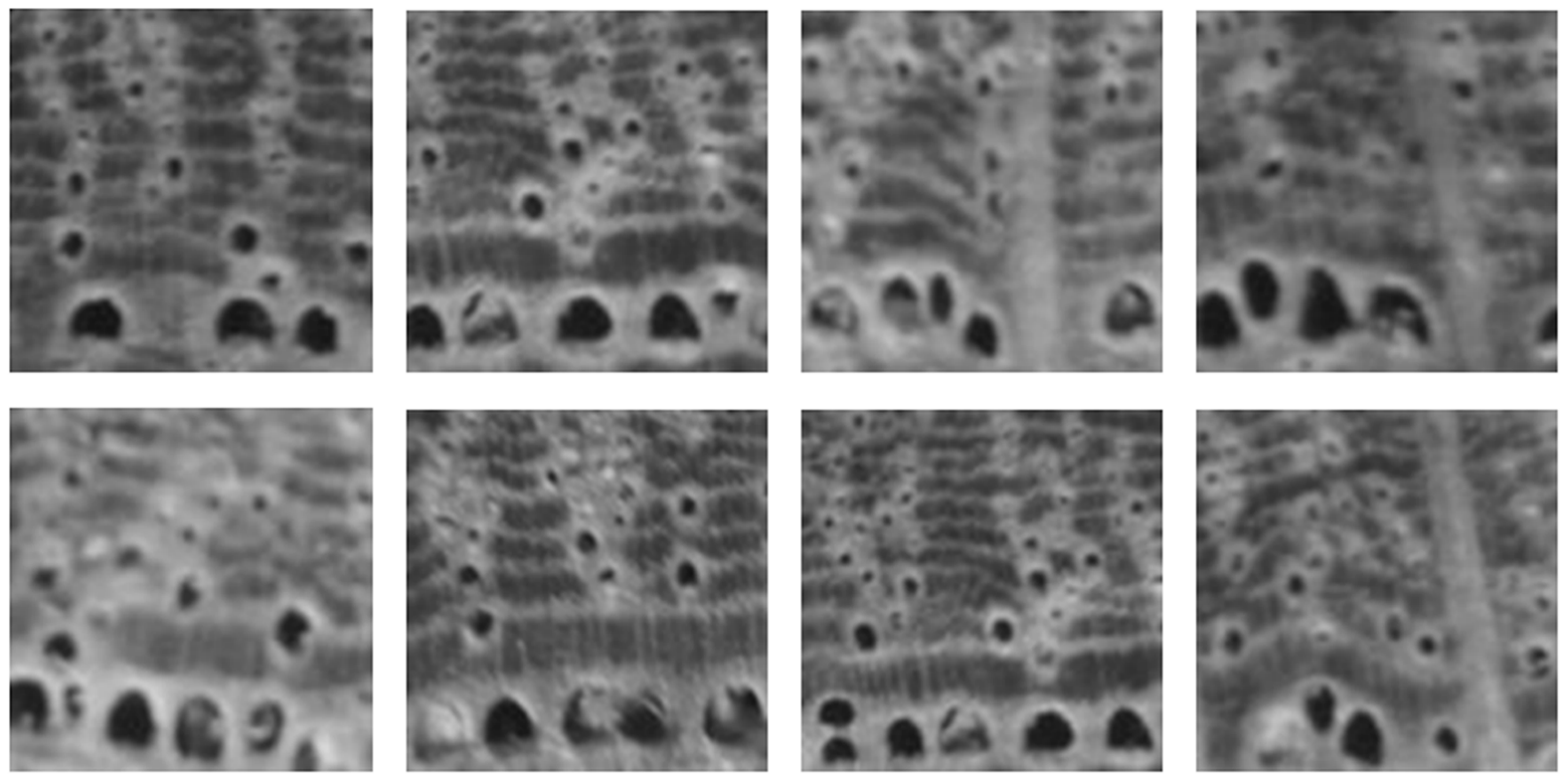
Eight images of a single wood species (*Quercus acutissima Carruth*).

Fig. 3 shows the wood species samples from the *ZAFU WS 24* dataset that is used in the following experiments. For comparison, the eight samples of a single wood species (*Quercus acutissima Carruth*) are shown in Fig. 4. From these two figures, we observe that visual features vary widely among wood species. These differences may be caused by variant sizes, density, arrangement, and distribution of the cellular organizations, such as pore, ray, and axial parenchyma within the wood. However, the regularity of these organizations within a given tree species enables wood experts to accurately identify wood species.

### 2.2 HLAC Based Method Overview

#### 2.2.1 Conventional HLAC feature

**Figure 5:**
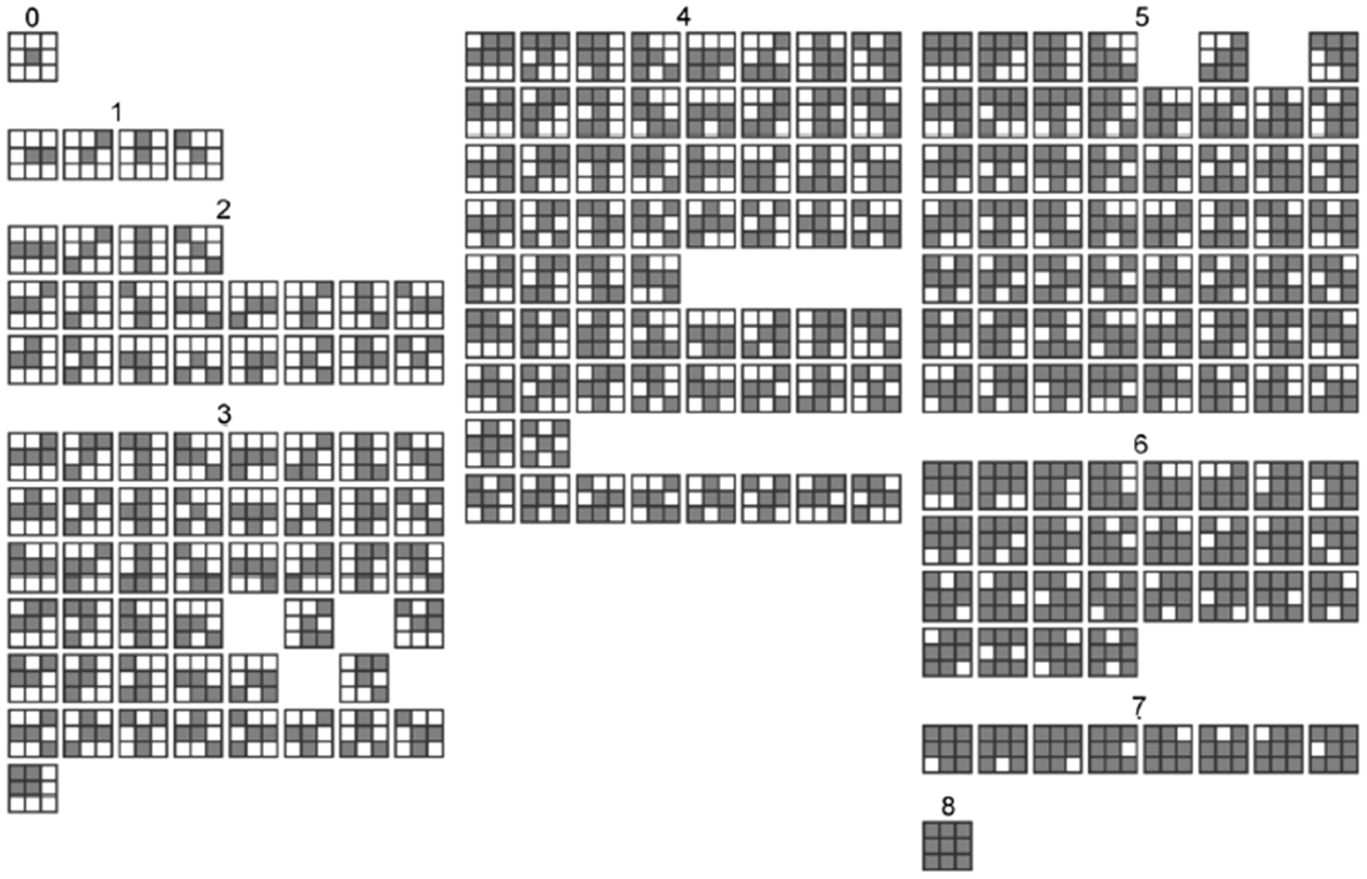
223 masks. (order 0–8; displacement 3×3 pixels) [34]

Otsu's HLAC feature is derived from *N*th-order autocorrelation functions, described as follows [22]: 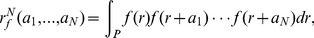
(1) where ***r*** is the image coordinate vector and ***a*** *_i_* are the displacement vectors; *f*(*r*) is the image intensity function on the retinal plane P. To realize efficient and effective feature evaluation, *f*(*r*) is restricted to binary images. Then, the *N*th-order autocorrelation function counts the number of pixels satisfying the following logical condition: 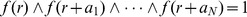
(2)To avoid the huge number of features captured by large displacement in higher-order autocorrelation, the original HLAC features are extracted within the region of a small local displacement (3×3) using second-order autocorrelation. The features are represented by 25 masks, the first 25 masks labeled with 0, 1 and 2, as shown in Fig. 5. Thus, the features are easily obtained by scanning these local masks over the binary image and summing the pixels that satisfy Eq. (2).

#### 2.2.2 Extending the HLAC Features

**Figure 6:**
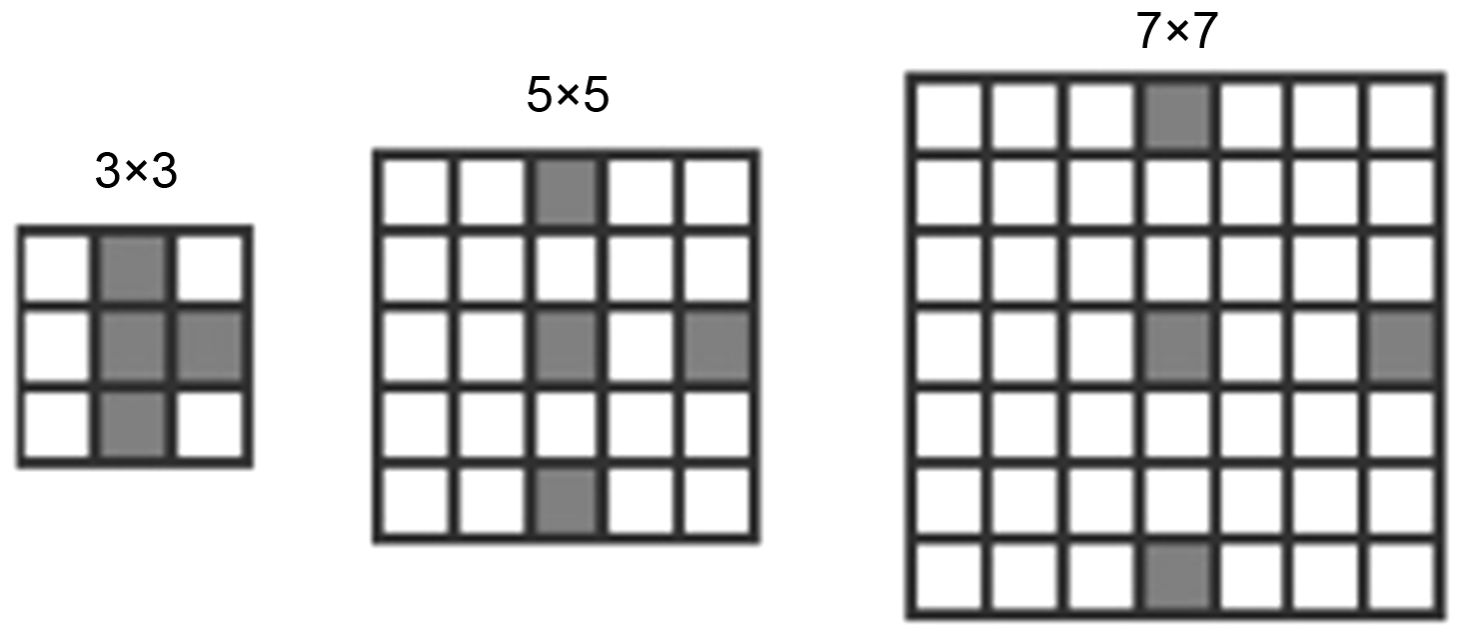
Larger masks obtained through dilation.

**Figure 7:**
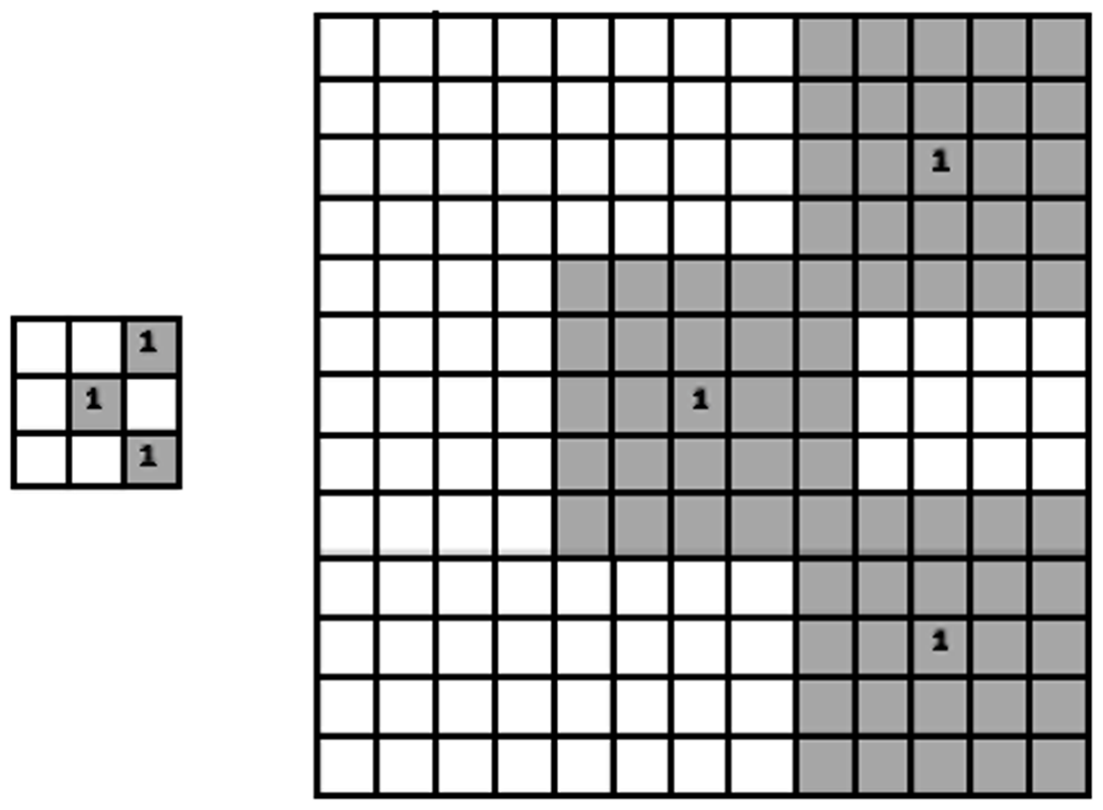
Large mask pattern realized by varying spatial interval.

To achieve a more detailed image characterization, we investigated various means of extending HLAC features. A direct approach is to increase the orders number. For example, Toyoda [33] extracted the features from 223 masks constructed from 7 orders, as shown in Fig. 5. Another extension is to enlarge masks to support large displacement regions (as shown in Fig. 6), and extracting the features with low resolution or low frequency. Although this approach enables extraction multi-resolution features from different sized masks, it ignores the variational regions among the reference points in the masks, and thereby introduces error. To reduce this error, Matsukawa and Kurita enlarged the mask pattern by expanding the reference points within the limit size (see Fig. 7) [35]. This approach restricts the displacement vectors *a_i_* in Eq. (1) to the subset: 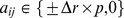, where p and Δ*r* are the pixel and spatial intervals, respectively. When autocorrelations in local regions are calculated at different Δ*r*, the HLAC feature becomes robust against small spatial difference and noise.

All of the methods for extending HLAC discused above seek detailed information from images with minimal computation by introducing larger masks while restricting the number of displacements. However, these methods cannot avert the problems caused by larger displacement and higher order autocorrelations in practical applications. Our proposed MMI method base on the mask matching technique, retains the rich texture information in the image. This information is conveniently embodied in the histogram. Nevertheless, the histogram method fails in many applications because it loses structure information of the object. In the next section, we introduce several statistical and geometric features unsed in our MMI approach. Unlike previous HLAC methods, which use a single feature (i.e. the number of mask matches), the MMI method allows features with larger displacement and higher order data, thereby enhancing the texture classification capability.

### 2.3 Feature Extraction from MMI

In this section, we discuss feature extraction from MMI in detail. MMI naturally retains the information obtained by HLAC. Therefore, the method inherits the desirable object recognition properties of HLAC, such as shift-invariance and additivity [22]. The MMI code can be downloaded from http://home.ustc.edu.cn/~hangjunw/code.htm.

#### 2.3.1 Definition of MMI

**Figure 8:**
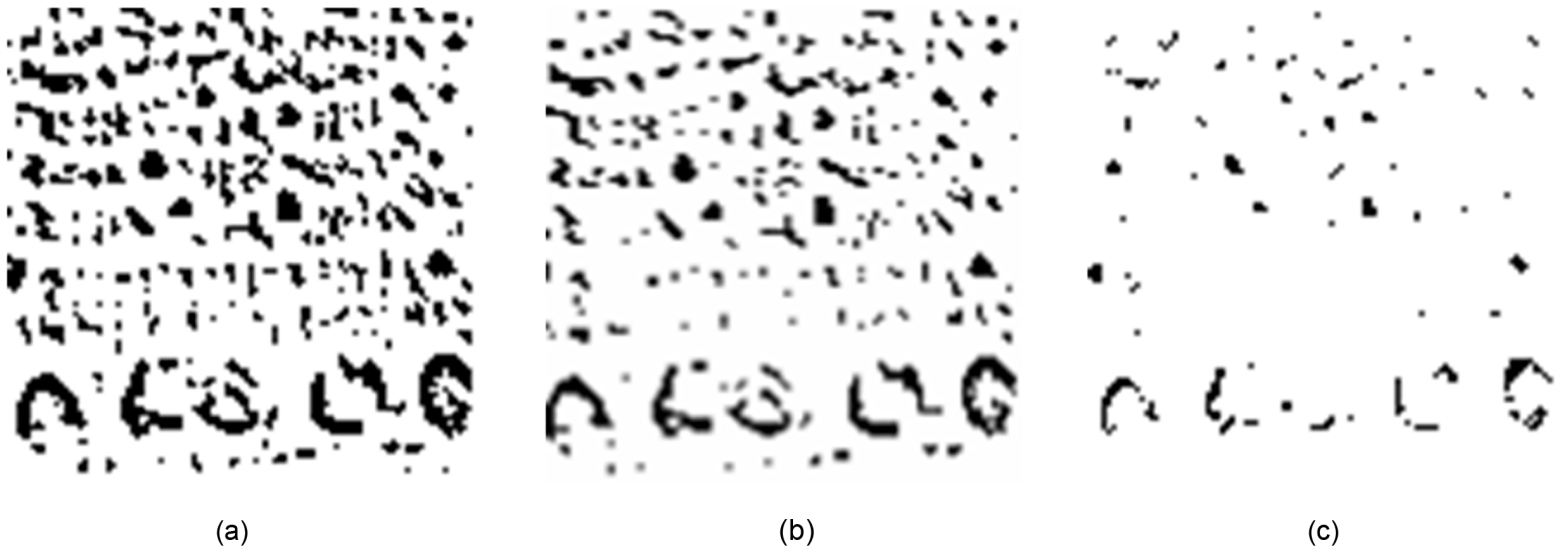
MMI implementation. : (a) Original image; (b) and (c) are MMI outputs of (a) using the 2^nd^ and 7^th^ masks in Fig. 5, respectively.

Let *f* be a binary image, and define ***r*** = (*x*, *y*)*^t^* as a position vector in *f*, where 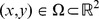. The MMI at pixel (*x*, *y*) for mask *i* is then defined as 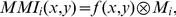
(3) where 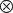 is an autocorrelation operation, *M_i_* is the *i*th mask, which may be ranked as shown in Fig. 5; *i* ∈ [1,*N*], and *N* is the number of the masks. The MMI outputs for the 2^nd^ and 7^th^ masks of Fig. 5 are illustrated in Fig. 8.

Conventional HLAC methods count only those masks that match the original image (for binary images) or sum the products of the corresponding pixels intensities (for gray images). In MMI, we may define several statistical and geometric features, such as those presented by Chen in [38], to obtain more useful information from images for classification.

#### 2.3.2 Statistical features of MMI

Here we define the statistical features of MMI. Let 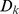 be a connected subgraph of 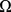 and |*D_k_*| indicate the area of the domain, where 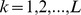; *L* is the number of connected subgraphs. For any 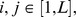 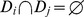. Thus we define five simple statistical features: 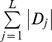, *L*, 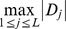, 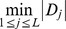, and 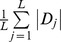. The first feature, 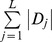, is adopted in existing HLAC methods. For classification purposes, these simple statistical features can be collated into a set of comprehensive features, denoted as simple statistical features of MMI (SSMMI). This feature set is expressed as follows: 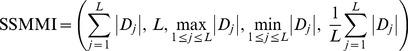
(4)


#### 2.3.3 Length histogram features of MMI

In addition to statistical features, MMI permits a number of geometric features. We present a new histogram feature, termed *length histogram* (*LH*), which combines the *width histogram* and *height histogram* to effectively represent geometric features. The geometric features of MMI are defined below.

Definition 1: Let *V* be the set of intensity values used to define adjacency, a term in graph theory that describes the relationship between two edges or vertexes. For a binary image, we set *V* = {1} if two adjacent pixels are labeled with 1. Thus, we define *h-adjacency* and *v-adjacency* as fellows:

1. *h-adjacency*: A pixel *p* at coordinate (*x*, *y*) and a pixel *q*, both containing values from *V*, are *h-adjacent* if the coordinates of *q* are (*x*+1, *y*) or (*x*−1, *y*).
2. *v-adjacency*: A pixel *p* at coordinate (*x*, *y*) and a pixel *q*, both containing values from *V*, are *v-adjacent* if the coordinates of *q* are (*x*, *y*+1) or (*x*, *y*−1).

Definition 2: A *horizontal* (or *vertical*) *line segment* from pixel *p* located at (*x*, *y*) to pixel *q* located at (*s*, *t*) is a sequence of distinct pixels with coordinates: 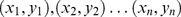
(5) where (*x*_1_, *y*_1_) = (*x*, *y*) and (*x_n_*, *y_n_*) = (*s*, *t*); (*x_i_*, *y_i_*) are *h-adjacent* and *v-adjacent* to (*x_i_*−1, *y_i_*−1), respectively, and *n* is the length of the line segment. Note that the values of other pixels adjacent to *p* and *q* are not members of V.

Definition 3: The *Width* (or *Height*) *histogram* is a function, *m_i_*, that counts the number of *horizontal* (or *vertical*) *line segments* of length *i* (also called bins). The graphical represntation is merely one way of representing a histogram.

**Figure 9:**
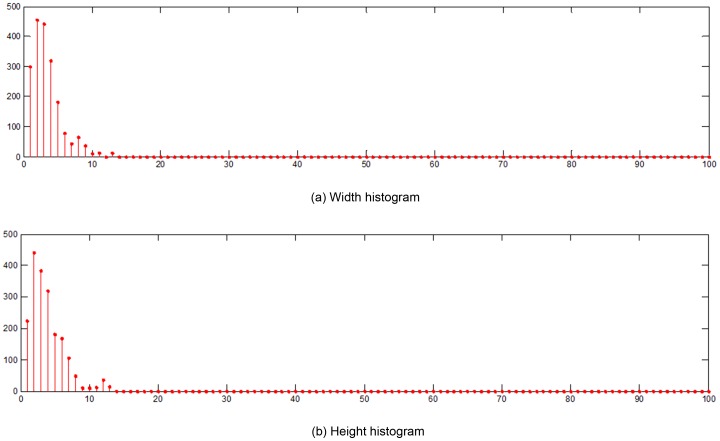
Example of length histogram of Fig. 8(b).

Width and height histogram of the image in Fig. 8(b) are shown in Fig. 9. The width and height of the image is 100 pixels.

## Results and Discussion

### 3.1 Experimental results on *ZAFU WS 24* dataset

The experiments in this study were conducted on grayscale dataset images. All experiments were implemented thirty times using randomly selected training and test images, and the average recognition rates was recorded for each run. The experimental results are reported as the mean and standard deviation of the thirty repeats. At last, the performances of the MMI features were assessed by two classifiers, k-Nearest Neighbors (kNN) and Support Vector Machine (SVM). All experiments yielded the same parameters: k = 1 with L1-norm distance (in kNN), and c = 100, d = 100, r = 1, C-SVC with linear kernel (in SVM, built from LIBSVM).

#### 3.1.1 Effects of the MMI and the mask number

**Figure 10:**
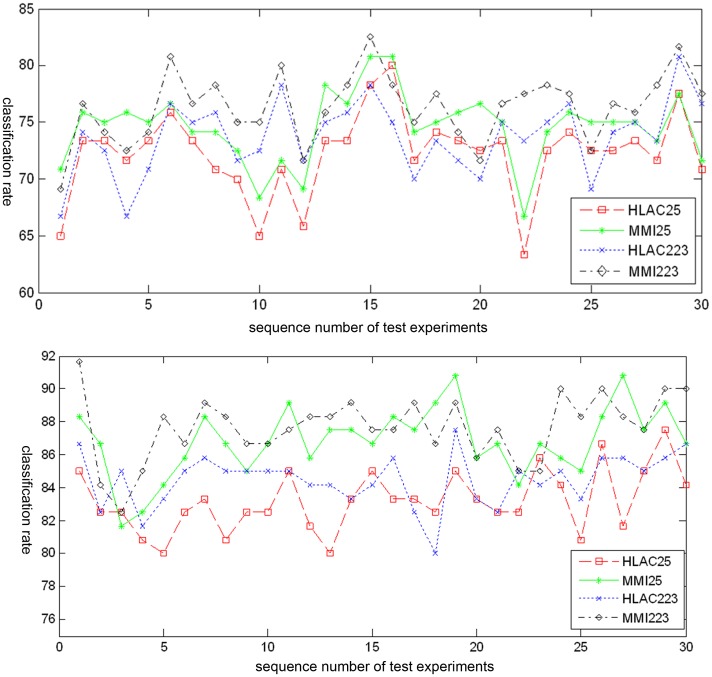
The experimental results of 30 test experiments.

First, the features recognition ability of MMI was compared for two mask groups, 25 and 223 (see Fig. 5). The SSMMI was computed from the five simple statistical features introduced in Section 2.3.2 (see Eq. (4)). The classification rates of 30 test experiments were evaluated by four methods: HLAC 25 (original HLAC with 25 masks), MMI 25 (MMI with 25 masks), HLAC 223 (original HLAC with 223 masks), and MMI 223 (MMI with 223 masks). The results of the kNN and SVM classifiers are shown in Fig. 10(a) and 10(b), respectively. Among 20 samples of each species, 15 were randomly selected as training samples, and the remaining 5 constituted the test samples. Evaluating the four methods on the same training and test samples, we obtained the means and standard deviations of the 30 experiments. The results are summarized in Table 1.

**Table 1:** Average classification results of HLAC and MMI using two mask groups and two classifiers.

Methods	kNN	SVM
**HLAC 25**	72.2222±3.6837	83.1944±1.8709
**HLAC 223**	73.6944±3.2730	86.8333±2.1150
**MMI 25 (SSMMI)**	74.5556±3.1618	84.4722±1.6153
**MMI 223 (SSMMI)**	**76.3333±3.0529**	**87.6667±2.0105**

From Fig. 10, we observe that the classification rates of fluctuate within a certain range throughout the 30 tests, regardless of methods. The classification rates may be affected by different training and test samples among different methods. Overall, however, the classification rates (from highest to lowest) is ordered as MMI 223>MMI 25>HLAC 223>HLAC 25 (see also the mean classification rates listed in Table 1). Also from Table 1, we observe that MMI outperforms HLAC for the same mask group; MMI 25 and HLAC 223 yield similar performances, while the performance of MMI 25 is about 0.86% higher than HLAC 223 in kNN, and 2.36% lower in SVM. On the other hand, the feature length of MMI 25 is much smaller than that of HLAC 223. These results suggest that the MMI features, more of which participate in classification than in HLAC, play a strong role in the classification process.

#### 3.1.2 Effects of statistical and geometric features of MMI on performance

In this experiment, we compare the performances among the statistical (SSMMI) and geometrical (LH) features of MMI. The performances are evaluated on two mask groups (25 and 223).

The experiments were performed under the conditions described in Section 3.1.1. The final results are listed in Table 2.

**Table 2:** Performance of MMI using different features.

Methods	kNN	SVM
**SSMMI 223**	75.0278±3.0987	86.7500±2.5366
**LH 223**	**83.8889±2.7624**	85.2778±2.8478
**SSMMI 25**	73.8056±3.1156	**86.8056±2.7602**
**LH 25**	82.4879±3.1543	86.5000±3.0669

As shown in Table 2, both SSMMI and LH yield high classification rates. Coupled to the SVM, the performance of SSMMI and LH are nearly identical; the former exceeds the latter by a mere 1.2222% and 0.0556% on 223 and 25 masks, respectively. However, under kNN, LH outperforms SSMMI by approximately 9%. In addition, the overall performance of LH is higher under kNN classification, indicating that kNN, as a simple and rapid classifier, is applicable to many real tasks.

The LH features are particularly advantageous because they are insensitive to the number of masks because of their extended order and displacement properties. Comparing 25 and 223 mask groups, the performances of the 4 MMI features methods in Table 2 are relatively close, and the LH classification improves with fewer masks. Thus, our method yields higher classification performance with smaller mask numbers, and thereby considerably reduces the spatio-temporal complexities of classification.

#### 3.1.3 Comparison of performances among different methods

In the third series of experiments, we compared our proposed MMI methods with recently proposed HLAC based methods: namely, GLAC [32] and HLACLF [39]. GLAC uses spatial and orientational auto-correlations of local gradients to extract richer structure information from images and obtain more discriminative power than standard histogram based methods, such as HOG [40] and SIFT [21]. Here, we adopted the parameters specified in [32] (for more details on GLAC, refer to [32]). In addition, HLACLF can extract the features from grey-scale images using different masks, allowing closer image analysis from the information of two-dimensional distributions, as well as the directions information. To realize a consistent comparison among the methods, we assume that images are never spatially blocked. The results of this experiment are listed in Table 3.

**Table 3:** Comparison of MMI with GLAC [32] and HLACLF [39].

Methods	kNN	SVM
**SSMMI 25**	73.8056±3.1156	**86.8056±2.7602**
**LH 25**	**82.4879±3.1543**	86.5000±3.0669
**GLAC**	78.4722±3.5765	84.3611±2.9660
**HLACLF 25**	54.8056±4.8625	69.0833±4.2792
**HLACLF 223**	46.4444±5.0663	68.5000±3.9172

According to Table 3, the proposed MMI methods outperform GLAC and HLACLF with one exception: GLAC outperforms SSMMI 25 under the kNN classifier. Here, the performance of GLAC is similar to that of MMI because GLAC extracts features from image gradients, which better describe image characteristics. However, large differences appear between MMI and HLACLF. Similar to 2DPCA [41], the later method extracts features from the 2-D direction of patterns, and is essentially equivalent to the line blocked HLAC method [39]. LH 25 outperforms HLACLF 223 by 36.0435% under the kNN classifier, while SSMMI 25 outperforms HLACLF 223 by 18.3056% under the SVM classifier.

Two major problems exist in HLACLF. First is the high grey value problem, which occurs when discriminative data in the grey images are overwhelmed by useless data in accumulation for the corresponding mask. The second problem related to image alignment. Wood data will not align with stored images because of inherent hashing of cell tissue around the tree rings, which complicates image preprocessing even in the same wood species. Nevertheless, the MMI methods can prevent both problems, since they impose no directional constraints on feature extraction. Instead, the features are identified from semantic object formed after threshold or edge operation. Those characteristics of MMI offer a distinct advantage.

### 3.2 Discussion

#### 3.2.1 Further Interpretations of MMI

By conducting experimental on the *ZAFU WS 24* dataset (see Section 3.1), we have verified the strong performance of our methods, on account of the advantageous features of MMI. Here, we illustrate these features with some simple visual examples.

**Figure 11:**
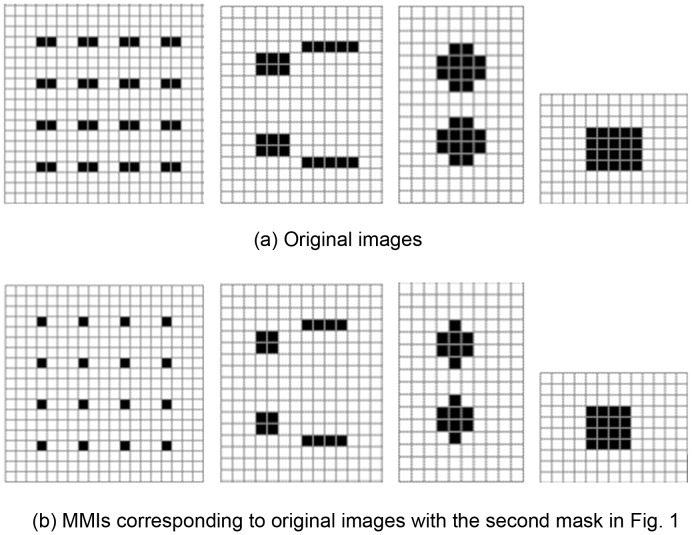
Comparison of different classes of original and MMI images.

**Figure 12:**
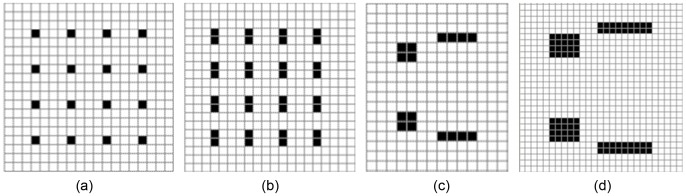
Comparison of identical image classes on mask matching images.

**Figure 13:**
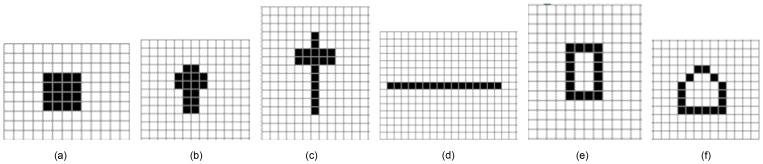
Different configurations of MMIs containing 16 pixel points.

1. Comparison of different classes of images.
  First, we selected four image classes containing very different shapes, as shown in Fig. 11 (a). The MMIs corresponding to the images, using the second mask in Fig. 5, are shown in Fig. 11 (b). From this figure, we observe that the conventional HLAC cannot distinguish among the four image classes (all HLAC features sum to16). However, by virtue of the number of the connected subgraphs *L*, MMI clearly distinguish the image classes (yielding corresponding values of 16, 4, 2, and 1). Moreover, the maximum and minimum values of the connected subgraphs, 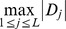
and 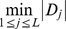 in SSMMI (which are identical in this case, being 1, 4, 8 and 16, respectively, for the four image classes), are also readily distinguish among the four classes. Consequently, by introducing MMI features, the method guarantees that different image classes yielding the same mask count contain different features, which increases the discriminated capability of the method.
2. Comparison of the same classes of images.
  Here we further discuss the case of two identical image classes. The images shown in Fig. 12 (a) and (b) are the mask matching images of one class, while those in Fig. 12 (c) and (d) belong to a separate class. The mask counts, i.e. the sum of the products corresponding to the second mask of the image, are 16, 32, 16, and 76, respectively for the four images. The same class may yield different mask counts; conversely, different classes may yield the same count. This situation is averted by introducing the MMI features. For example, in Fig. 12, although the mask count varies widely within a class, the masks contain the same number of connected subgraphs. Thus, the MMI method is superior to exsiting method at identifying such tasks. And the MMI feature also is a type of scale invariant feature.
3. Effects of length histogram.
  In general, the length histogram obtains geometric information that can distinguish among objects of various shapes. An example is shown in Fig. 13. The mask count of all 6 images is 16. However, the 6 images vary significantly in shape. These shape differences are difficult to distinguish using the SSMMI features defined in Eq.(4). However, from the length histogram features, statistical structure information of the image can be obtained from both horizontal and vertical directions. For instance, the width histogram features of the 6 images in Fig. 13 are (4,4); (2,4) (4,2); (1,8) (5,2); (16,1); (1,8) (4,2); (1,8) (2,2) (6,1), respectively. Here, the image features are separated by a semicolon. The first and second components in each parenthesis denote the width and width count, respectively. All features yielding zero count are omitted from the width histogram. This histogram provides an intuitive means of identifying differences among these six images. The height histogram possesses similar characteristics. Therefore, image classes of different textures are readily distinguished by the length histogram.

#### 3.2.2 Properties of Length Histogram

We have demonstrated the efficacy of our proposed MMI method as a classification system. Intuitively, MMI method is superior to HLAC because it extracts more features that are relevant to the classification process. In fact, the excellent properties of length histogram (LH) render it suitable for a wide range of image texture analyses.

**Figure 14:**
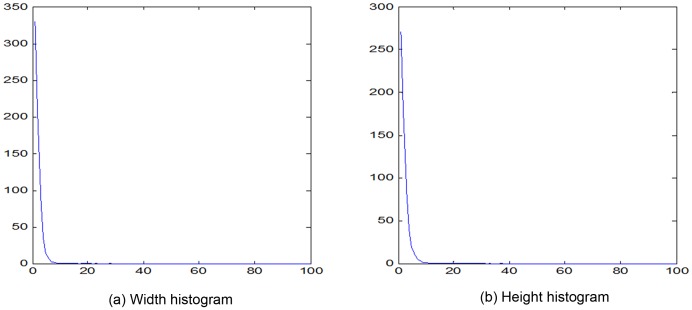
Example of length histogram (average of all samples).

1. Some structural information retained in LH. Although the applicability of the standard histogram method is limited by loss of object structure information, the length histogram proposed in this paper contains partial structural information of the image. This is achieved by counting the number of two co-occurrence pixels separated by a certain distance, which captures geometrical characteristics of the objects appearing in the image. Thus the structure information of object is preserved in the LH.
2. Extending the order and displacement region of HLAC. The LH features in MMI is equivalent to counting the number of the matched pattern masks with high-order and large displacement. Thus, the order and displacement of HLAC can be gradually enlarged until the mask size reaches the width and height of the image. However, this approach is suitable only if the reference points in the mask are arrayed in the continuous horizontal or vertical direction (see masks 2, 4, 6, 8, in Fig. 5).
3. Simple and efficient algorithm. Because the length histogram requires a simple computation, it considerablely reduce the computational cost of MMI method Regardless of the width or height of the histograms, provided that the image is scanned only once, we can obtain the frequencies of various lengths of line segments in the image. Therefore, the efficiency of the new feature extraction algorithm is proportional to image size (the total number of pixels in the image). Since the proposed method has proven effective and efficient in texture classification and recognition, it is eminently suitable for practical applications.
4. Sparse property. The MMI length histogram is generally sparse. The length histogram shown in Fig. 14 is an average length histogram of all sample pictures used in our experiments. We note that most of the bins are empty, and that the non-zero values are concentrated in a small portion of the lengths (bins). This result indicates that the obtained LH features are very sparse indeed. Thus, MMI method can reduce the spatio-temporal complexity of the problem, allowing more time for processes such as transformation and classification.

## Conclusions

Wood recognition is critically important for wood industries and sciences because it clarifies the anatomic features and properties of wood, which determine how the wood species is used. As a new research area, wood recognition remains particularly challenging in computer vision (CV) and pattern recognition (PR) fields. Because trees are found throughout a wide range of natural environments, their wood features are highly variable. Such variation can frustrate many identification procedures, and confuse classification attempts by the apparent lack of consistency. This paper presents a new efficient method for wood recognition based wood stereogram images, called *Mask Matching Image* (MMI). The method, an extension of HLAC, resolves the problems inherent in HLAC by incorporating both statistical and geometrical features. In particular, the length histogram embodies both width and height histograms. This method enables the extraction richer information from images with greater discriminative power than is possible using previous HLAC-based methods. We have confirmed the efficacy of MMI in extracting local texture information, which is required for activities such as texture classification, face recognition, and gait recognition.
